# Grave Cardiac Symptoms Due to Multiple Dental Abscess
1Read at a Meeting of the Bristol Medico-Chirurgical Society, February 13th, 1924.


**Published:** 1924-04

**Authors:** W. R. Ackland

**Affiliations:** Hon. Consulting Dental Surgeon, Bristol Royal Infirmary


### GRAVE CARDIAC SYMPTOMS DUE TO MULTIPLE DENTAL ABSCESS.[1]

BY

W. R. ACKLAND, M.D.S.,

*Hon. Consulting Dental Surgeon, Bristol Royal Infirmary.*

FORTY years ago, when I was at the Hospital, there was very little talk of oral sepsis or pyorrhœa, and all the teaching was directed to the saving of teeth at all costs. Consequently, owing to the fixity of these early impressions, I find myself approaching all those cases attributed to oral sepsis with an instinct of suspicion and possibly of doubt. But the cases that have happened in my own practice, together with the work of such men as Dr. William Hunter and Sir William Willcocks, have made me feel that I have leaned possibly too much to saving teeth. All the same, I am convinced that a great deal too much has been done in the other direction.

[1] Read at a Meeting of the Bristol Medico-Chirurgical Society, February 13th, 1924.

I am bringing to your notice to-night a case which I am bound to say has convinced me, so to speak, against my will, that the condition of the teeth was the cause of the heart symptoms. The patient is himself a doctor, and is here to-night to bear out my statement of his case.

His age is 42, had never previously suffered from cardiac symptoms. On April 23rd, 1923, he had acute coryza with mild laryngitis, diagnosed as influenza, and was in bed for two weeks. During this illness he had occasional precordial pain and sensations of fluttering. He attributed these symptoms to two toxins—influenza and nicotine, and ceased smoking. In spite of this, the cardiac symptoms persisted during the next two months, and increased in severity. He was easily tired, and had mild dyspnœa after slight exertion. On June 9th, 1923, he consulted a colleague, who diagnosed post-influenzal myocarditis. At 10 p.m. on June 15th he had a severe rigor, but the temperature remained normal. The next morning he had pains in both knee-joints and in the left wrist. At 11 p.m. on June 16th he had severe cramp of the recti abdominales muscles, followed bysweating. When he awoke on the morning of June 17th there was considerable cardiac distress—pain, irregularity, and sensations of impending dissolution. The patient was seen by Dr. Farrer Thompson, of Woburn, who found the pulse rate 60, irregularly intermittent. The area of cardiac dullness was not increased, but the first sound was very faint, and the second was accentuated and reduplicated. Extra-systoles were present, and the systolic blood pressure had fallen to 100 m.m. Hg. The tongue was thickly coated, and the patient was perspiring. Dr. Farrer Thompson diagnosed toxic myocarditis, and advised the patient that pus was probably present, as the previous influenza could not account for the present toxic condition. He advised that the teeth should be X-rayed in the first place. If no sepsis were found, the accessory sinuses should be carefully examined. The teeth were X-rayed, with the following result: Four dead teeth were discovered, with three chronic abscesses and a certain amount of pyorrhœa alveolaris.

After the first extractions the cardiac symptoms began to disappear, and the patient decided to make a "clean sweep," so we gradually extracted the whole of his teeth during July and August, and ultimately fitted dentures.

The cardiac symptoms have entirely gone, and the general health is better than it has been for five or six years.

### DISCUSSION.

Dr. X. (the patient) said that he wished to add something to the account given by Mr. Ackland. He had for over two years suffered chronic ill-health; quite two years ago he had had a severe "rheumatic" attack with rigor, precordial pain, joint pains, and sour sweat, followed by hæmaturia and swelling of the ankles. He emphasised the importance of making a definite diagnosis in cases of disordered cardiac action.

Dr. P. WATSON-WILLIAMS urged the importance of considering all possible sources of sepsis—teeth, tonsils, bowels, accessory nasal sinuses or others. Often the foci of infection that were least conspicuous as sources of visible pus were the most dangerous to the patient. In such cases a difficulty in mental concentration was frequently an early and important sign.

Dr. HERAPATH questioned the diagnosis of myocarditis. There appeared to be no tachycardia, and no dilatation of the heart in the case, which did not fit in with that diagnosis. Disordered action of the heart was now thought by many to be due to influence of the nervous system on the heart, especially probable if fear were present. Often there was no illness preceding the disordered cardiac action.

Dr. CAREY COOMBS said that he had investigated the importance of dental sepsis. There appeared no evidence in his series of cases that naked-eye dental sepsis commonly affects the heart.

Dr. X. in reply said that his cardiac condition appeared not to have been made quite clear. His pulse was 130, irregular, and with extra systoles; the apex beat, however, was not displaced. He had not observed any condition of fear before or since the onset of his trouble. He had, however, noticed that mental concentration was increasingly difficult. He gave an amusing description of spa therapy, especially with "radium" water. In conclusion, he wished to thank Mr. Ackland for the remarkable success of his treatment, and to emphasise the value of skiagraphy in assisting the diagnosis.

